# Impact of exotic macroalga on shorebirds varies with foraging specialization and spatial scale

**DOI:** 10.1371/journal.pone.0231337

**Published:** 2020-04-10

**Authors:** Alice F. Besterman, Sarah M. Karpanty, Michael L. Pace

**Affiliations:** 1 Department of Environmental Sciences, University of Virginia, Charlottesville, Virginia, United States of America; 2 Department of Fish and Wildlife Conservation, Virginia Tech, Blacksburg, Virginia, United States of America; CSIRO Townsville Australian Tropical Sciences and Innovation Precinct, AUSTRALIA

## Abstract

Exotic species may increase or decrease native biodiversity. However, effects of exotic species are often mixed; and indirect pathways and compensatory changes can mask effects. Context-specific assessments of the indirect impacts of exotic species are also needed across multiple spatial scales. *Agarophyton vermiculophyllum* (previously *Gracilaria vermiculophylla*), an exotic, invasive macroalga, has established throughout the western hemisphere with reported positive or neutral impacts on biodiversity. Shorebirds are an important group for conservation in areas invaded by *A*. *vermiculophyllum*. We assess the impacts of this invader on shorebirds by measuring behavior and habitat selection at spatial scales ranging from algal patches to the entire study region. Birds were considered either flexible-foragers that used diverse foraging techniques, or specialized-foragers that employed fewer, more specialized foraging techniques. Responses were scale dependent, with patterns varying between spatial scales, and between behavior and habitat selection. However, a general pattern of habitat selection emerged wherein flexible-foraging shorebirds preferred *A*. *vermiculophyllum* habitat, and for specialized-foragers, habitat selection of *A*. *vermiculophyllum* was mixed. Meanwhile, flexible-foraging birds tended to neutrally use or avoid uninvaded habitat, and specialized-foraging birds mostly preferred uninvaded habitat. Shorebird behavioral response was less clear; with flexible-foragers spending less time on bare sediment than expected, the only significant response. Shorebird response to *A*. *vermiculophyllum* differed by foraging mode; likely because flexible, opportunistic species more readily use invaded habitat. Increases in *A*. *vermiculophyllum* could result in functional homogenization if the bare habitat preferred by specialized-foragers is reduced too greatly. We hypothesize the effect of scale is driven by differences among tidal flats. Thus, tidal flat properties such as sediment grain size and microtopography would determine whether foraging from *A*. *vermiculophyllum* was optimal for a shorebird. Specialization and spatial scale are important when assessing the biodiversity conservation impacts of invasive *A*. *vermiculophyllum*.

## Introduction

Despite abundant evidence of catastrophic ecosystem change from species invasions, exotic, invasive organisms in some cases have positive effects on ecosystem function and biodiversity [1–4]. Exotic species that modify physical structure of environments exhibit bimodal impacts, depending on whether they increase or decrease habitat heterogeneity [1,3]. When exotic species add complexity or compensate for lost habitat complexity, the density and diversity of native biota increase [1,3–7]. As heavily human-modified ecosystems become more common in the 21^st^ century, invasive species may provide conservation opportunities [1,3].

Still, detectable positive impacts of invasives are context-specific since factors such as spatial scale and life history are strong mediators [1,8]. Positive impacts for one species or guild may indirectly cascade to negatively affect another group of native organisms [9]. A patchily distributed invasive species may locally decrease abundance and richness of native species, but increase diversity at the landscape scale [1]. Specialized species may respond negatively to altered ecosystem function caused by exotics, even as total species richness and density increase [1,2]. The loss of specialists can result in functional biotic homogenization, where guilds of species that perform specialized functions are lost from an ecosystem [10]. However, detecting effects of invasive species, per se, is difficult due to indirect, compensatory, and long-term responses in native communities [9,11]. As a result research on invasive species must be carefully contextualized [1,9,11].

Benthic macroalgae are increasing globally due to both eutrophication and species invasions [2,12]. In some cases invasive macroalgae have devastated benthic communities and water quality [2]; however, most exotic macroalgal species have mixed effects depending on the conditions in the invaded ecosystem and the response variable considered [2]. *Agarophyton vermiculophyllum* (formally known as *Gracilaria vermiculophylla*), a cryptically-invasive, habitat-modifying seaweed from east Asia, has established populations throughout the western hemisphere, and since discovery its effects have been perceived as either neutral or “positive” for coastal ecosystems [4,5,7,13,14]. However, examples of negative effects have been documented in particular cases, including seagrass under high temperature and settlement on oysters [15,16]. Thick accumulations of *A*. *vermiculophyllum* occur on tidal flats, which are otherwise unvegetated, or sparsely vegetated, soft-sediment, low relief intertidal environments. *A*. *vermiculophyllum* is facilitated by a tube-building polychaete, *Diopatra cuprea*, that incorporates thalli into its tube and stabilizes macroalgal mats [17]. In these largely homogeneous environments *A*. *vermiculophyllum* provides ecosystem services, like wave-attenuation, and increasing the abundance of benthic macroinvertebrates including the economically important *Callinectes sapidus* [4–6]. *A*. *vermiculophyllum* is not grazed by macroinvertebrates in most invaded estuaries, so it provides structural rather than direct nutritional benefits [18,19]. Recently however, claims that *A*. *vermiculophyllum* is an invasive species with positive effects on many native species, have been challenged as premature. While in some cases *A*. *vermiculophyllum* has had apparently positive effects, the number of understudied responses combined with some observed negative impacts indicate further research on impacts of this species is needed [20,21].

Shorebirds are one group of organisms that extensively use tidal flats where they forage for benthic invertebrates often in dense aggregations [22]. Globally shorebirds are declining, and many species are protected or considered in conservation management plans [23–25]. *A*. *vermiculophyllum* may have indirect impacts on these native predator communities transmitted by their invertebrate prey living on and in marine sediments. Many shorebird species have diverse diets, consisting of marine worms, small crustaceans, gastropods, bivalves, and insect larvae. Diets are often regionally specific, with shorebirds focusing on the most abundant prey in a given location [26–30]. Macroalgal mats can have context-specific effects on macroinvertebrates. Some epifauna, such as small crustaceans and snails, may become more abundant in response to *A*. *vermiculophyllum* invading. However, epifaunal prey may be less accessible due to macroalgae obscuring visual foraging cues for shorebirds. Infaunal changes vary depending on whether animals are surface-deposit feeding or subsurface-deposit feeding. Surface-deposit feeding polychaetes typically reliant on oxidated, bare sediment, may move to shallower depths to avoid anoxic conditions, or when oxygen is reduced more severely, their abundances may decline. Subsurface-deposit feeders are less likely to be affected; however, these deeper prey are less important for many short-billed shorebirds that are limited to feeding on shallow, surface-deposit-feeders and epifauna. The impact of *A*. *vermiculophyllum* for foraging shorebirds depends on their dietary flexibility, and the magnitude of the changes to the abundance and distribution of the prey community [4,5,31,32]. Observing shorebird distribution and behavior may provide a clearer, more integrated picture of how *A*. *vermiculophyllum* affects prey resources than monitoring invertebrates directly.

Studies of shorebird predator responses to *A*. *vermiculophyllum* are limited. In one recent study, Haram et al. 2018 observed that shorebird density increased in response to *A*. *vermiculophyllum* in the southeastern U.S [7]. Likewise, shorebirds reached peak density on mats of *Gracilaria* spp. in the northwestern U.S. [33], although in that case the macroalga was not identified to species, it would likely be similar morphologically to *A*. *vermiculophyllum*. However, some species avoided *A*. *vermiculophyllum* mats in the southeastern U.S. [7]. These species may avoid *A*. *vermiculophyllum* because they are more specialized-foragers that cannot optimally forage from macroalgal mats [31,34]. Theory and empirical evidence suggest specialists will respond negatively to invasive species, while generalists either maintain or increase their populations [8]. The response of native biodiversity to the invasion of *A*. *vermiculophyllum* along a gradient of specialization has not yet been addressed, and is important because globally specialist species have experienced greater declines as a result of anthropogenic disturbance when compared to generalist species [10,35]. In order to understand the full conservation implications of the *A*. *vermiculophyllum* invasion explicit differences in the response of specialist and generalist predators needs evaluation.

The goal of this research was to examine how species foraging specialization mediates the indirect effect of an exotic species on the biodiversity of native predators. We addressed this question in an *A*. *vermiculophyllum*—shorebird system by quantifying shorebird foraging behavior and habitat selection [36]. Many shorebird species are opportunistic foragers with respect to diet [27,30]. But foraging specialization can occur as a result of morphology [37,38], diversity of prey capture techniques employed [39], and whether organisms are ‘continuous’ or ‘sit-and-wait” predators [40], even while the diversity of prey taken is high. These traits can result in birds preferentially using certain types of substrates, or microhabitats, where they have the greatest success given their foraging mode [37,39]. Therefore, when considering the effects of *A*. *vermiculophyllum* on shorebirds, the most important traits to consider would be foraging mode, rather than diet. Macroalgae change the structure of substrate [19], may obscure visual foraging cues [31], and alter prey distributions [4,32]. It is possible shorebirds with specialized foraging modes for soft muds would be unable to optimally forage from the vegetated microhabitat created by *A*. *vermiculophyllum*. Meanwhile, shorebirds with flexible foraging modes that can use a diverse suite of foraging techniques might adapt their behavior to use this “novel”, invasive habitat. Because the response of individual organisms and populations to exotic species can vary with spatial scale [1,8], we investigated both foraging behavior and habitat selection of *A*. *vermiculophyllum* by shorebirds across multiple spatial scales. Whereas other studies have explored shorebird response to *A*. *vermiculophyllum*, this study is unique in its exploration across multiple spatial scales, and explicitly testing specialization as a driver of native species response. We investigated four main questions. First, do shorebirds selectively use macroalgal and bare (uninvaded) habitat? Second, do ‘specialized-foragers’ respond differently to *A*. *vermiculophyllum* than ‘flexible-foragers’? Third, do patterns of selection change with spatial scale? Fourth, does shorebird foraging behavior (proportion of time spent on different habitats, or time budgets) differ from population-scale habitat selection? We hypothesized that flexible-foragers would either prefer, or neutrally use novel macroalgal habitat, while specialized-foragers would avoid them. We also hypothesized birds would be less selective locally as compared to large spatial scales, and that birds would be more flexible in their foraging behavior than habitat selection.

## Methods

### Ethics statement

Approval for research activities and site access were acquired from the proper authorities. Our strictly observational work did not require animal care/use approval from the University of Virginia Animal Care and Use Committee. Sites included privately leased, privately owned, and public areas. Privately leased tidal flats are owned by the Virginia Marine Resource Commission (VMRC); permission was obtained from the VMRC as well as each individual lease holder. VMRC owned tidal flats that were not leased were also used, and we received permission to work on these areas. We received approval for all research activities conducted on VMRC property. The Nature Conservancy (TNC) privately owns some of the sites used, and we received permits to work on these areas, had our research methods approved by TNC, and followed all permit requirements. We collected data from Assateague National Seashore, and received a permit from the National Park Service (NPS) to conduct research on that site. All activities were approved by NPS, and we followed all permit requirements. We did not collect any protected macroinvertebrate species as a part of this study. Some species of shorebird observed in this study are listed as threatened. Proper distances and sampling protocols to avoid disturbing species were followed, and approved by TNC, and NPS. Only NPS used a numerical permit system, permit number: ASIS-2018-SCI-0005.

### Study system and species

#### Study area

**Fig 1:**
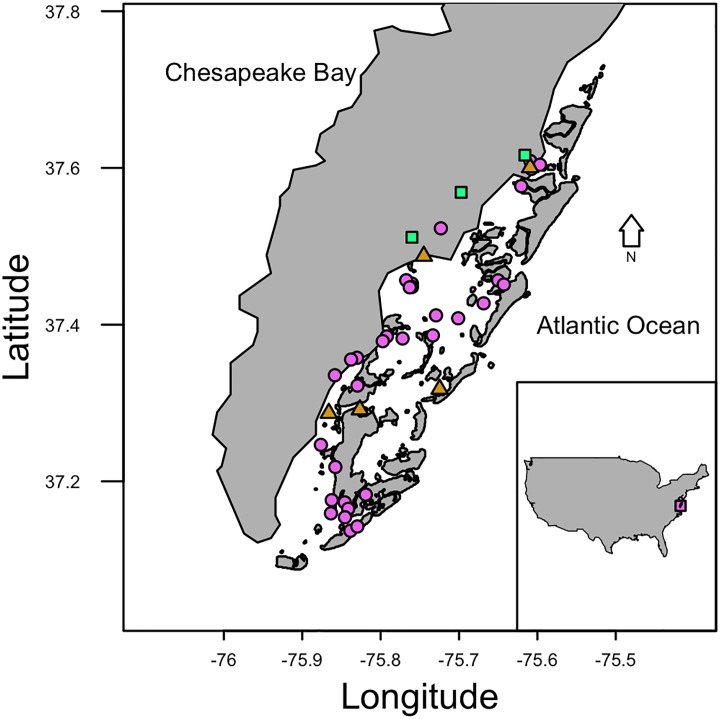
Study sites. Map of the Virginia Coast Reserve (VCR) with survey sites from 2016 and 2018. Land is shown in gray and water in white. Orange triangles = 2016 only. Purple circles = 2018 only. Blue-green squares = 2016 and 2018. Larger map is the VCR. Inset shows United States, and small violet box shows the boundary of the study region. One site is not shown because it was just north of the reserve boundary.

This work was carried out in the Virginia Coast Reserve Long-Term Ecological Research Site (VCR), a 110 km stretch of protected coastline including coastal bays, marshes, barrier islands, and tidal flats (Fig 1). This system is low nutrient, mostly undeveloped, and sparsely populated, allowing us to examine the effects of the exotic, invasive *Agarophyton vermiculophyllum* in isolation of common environmental changes related to human activity. The VCR is an important spring stopover for shorebirds during their northward migration [41]. Hundreds of thousands of birds stop to feed and replenish energy reserves in the VCR between April and May each year, and tidal flats are an especially important habitat for these birds.

In this paper we refer to ‘macroalgal’ habitat generally, but *A*. *vermiculophyllum* dominates macroalgal abundance with other species making minor contributions [42,43]. There is moderate macroalgal diversity in the VCR, and macroalgae are distributed throughout intertidal and subtidal habitats [42]. Mats are often interspecific, but *A*. *vermiculophyllum* accounts for > 65% of macroalgal biomass [43]. Mats of macroalgae grow over 10–40% of exposed area on tidal flats with 18 g dw m^-2^ average biomass, and maximum measurements of > 300 g dw m^-2^ [43]. Macroalgal mats are distributed in patches on tidal flats, and patches range in size from < 0.25 m^2^ to hundreds of m^2^. Further details describing macroalgal diversity and invasion in the VCR are presented elsewhere [13,42,43]. Mats are often interspecific, and we could not control for differential use of *A*. *vermiculophyllum* and native seaweeds.

#### Study site selection

We selected tidal flats for sampling that represented variability found in the reserve, and that were distributed from the mainland to the barrier islands (east-west) and along the entire north-south extent of the VCR (Fig 1). In 2016, nine tidal flats were sampled for shorebird use, macroalgal cover, and prey availability (Fig 1). Sites were defined as a 100 m radius semicircle (1.57 ha), with the plot edge defined by marsh or beach. Some plots were smaller because we did not have an unobstructed view of a 100 m radius semicircle. In 2018, we studied 36 sites using 60-m radius circular plots (1.13 ha). The 2018 sites that also spanned the entire VCR, and we sampled each for shorebird use and macroalgal cover (Fig 1).

#### Focal species

Seven shorebird species were selected for study. We conducted analyses first for each species, and then grouped by foraging mode. Shorebirds were assigned as flexible- or specialized-foragers based on the following functional traits: prey detection method, prey capture techniques, and bill morphology (Table 1). Many of these species are known to specialize on particular prey, differing by location [30,44–47]; however, little is known about diet during spring migration in the VCR. These species are all dietary generalists, focusing opportunistically on highly abundant and seasonal prey items [29,45–50]. The diets of these species significantly overlap [27], and the prey groups taken can occupy diverse habitats [51,52]. As a result, we did not sort shorebirds into specialization foraging-modes based on diet. Species-level responses were compared with those computed at the level of foraging mode to confirm the groupings.

**Table 1:** Focal species descriptions.

Taxa	Common Name	Mean (SD)	Prey Detection	Capture techniques	Bill Morphology	Foraging Mode
*Calidris alpina*	Dunlin	14.51 (28.8)	Tactile and visual	Probes, jabs, picks in substrate; surface tension feeds	Slightly decurved, bill length longer than head	Flexible
*Calidris*	Peeps	3.77 (14.5)	Tactile and visual	Pecks, probes, slurps/skims, surface tension feeds	Straight, thin bills approximately same length as head	Flexible
*C*. *pusilla*						
*C*. *minutilla*						
*Tringa semipalmata*	Willet	0.28 (0.7)	Tactile and visual	Diverse search- and -capture methods, e.g. pecks, probes, picks, plows, lifts	Straight bill much longer than head	Flexible
*Limnodromus*	Dowitchers	6.33 (20.7)	Mostly tactile, some visual	“Sewing-machine-motion”, jabs, probes	Straight bill much longer than head	Flexible
*L*. *griseus*						
*L*. *scolopaceus*						
*Pluvialis squatarola*	Black-bellied Plover	4.11 (10.3)	Obligate visual	Stop-and-run, pecks	Straight, heavier bill shorter than head	Specialized
*Charadrius semipalmatus*	Semipalmated Plover	2.61 (5.3)	Obligate visual	Stop-and run; Pecks, rarely probes	Very short, “stubby” bill	Specialized
*Numenius phaeopus*	Whimbrel	0.61 (1.1)	Mostly visual, some tactile	Stop-and-run; pecks or probes	Bill much longer than head, bill decurved	Specialized

Details on focal species studied in this paper. Mean density of birds per tidal flat plot (1.13 ha) and standard deviation (SD) are presented for 2018, n = 36 plots. Prey detection, capture techniques, and bill morphology of focal species described, and foraging mode of species either flexible or specialized designated.

Flexible-foragers included: *Calidris alpina* (dunlin), *Calidris spp*. (peeps), *Tringa semipalmata* (willet), *Limnodromus spp*. (dowitchers). We were unable to reliably differentiate between three species of *Calidris* sandpipers in the field. This group, peeps hereafter, was largely composed of *Calidris pusilla* (semipalmated sandpiper) individuals based on natural history, and documented sightings and range maps [47,53]. *Calidris minutilla* (least sandpiper) is also present in the system during spring migration, though significantly less abundant than semipalmated sandpiper [47,53,54]. It is possible but rare for *Calidris mauri* (western sandpiper) to use the VCR, although their appearance is mostly restricted to fall migration [53,55]. Similarly, *Limnodromus griseus* (short-billed dowitcher) and *Limnodromus scolopaceus* (long-billed dowitcher) could not be distinguished in the field. However, this group was likely composed of mostly short-billed dowitcher because long-billed dowitchers are very rarely observed in the eastern U.S. flyway during spring migration [56]. Birds considered flexible-foragers had comparatively straight, and long bills (Table 1). These bills allow birds to access deep resources and diverse substrates, and are associated with tactile foraging [37,38,46–49]. Flexible-foragers also used more capture techniques than specialized-foragers; for example, probing, pecking, and surface-tension feeding, as well as the ability to forage visually or tactilely.

The specialized-foragers were: *Pluvialis squatarola* (black-bellied plover), *Charadrius semipalmatus* (semipalmated plover), *Numenius phaeopus* (whimbrel) (Table 1). Specialized-foragers’ bill morphology physically restricted the types of capture techniques that could be used, and substrates accessed [29,37,38,44,45,50]. In the case of semipalmated and black-bellied plovers, birds have short and “chunky” bills that restrict the depth birds can forage from in sediments [29,38,50]. Whimbrel have very long, but sharply decurved bills. Decurved bills confer a number of advantageous for foraging, but are highly specialized for visual foraging on soft mud, and extracting worms and crabs from burrows [37,44,45]. Both plovers are obligate visual foragers, and employ only one or two capture techniques including the “stop-and-run” technique (Table 1). The stop-and-run technique refers to a search and capture technique where birds scan for prey while moving, then stop to capture prey [29,38,50]. Whimbrel forage almost entirely visually, usually using the same stop-and-run technique as plovers [44]. We inspected data prior to combining species to make sure patterns were not driven by only a few species, or divergent patterns were masked in the groupings.

#### General approach

We investigated whether shorebirds prefer or avoid macroalgal mats using surveys of shorebird abundance and habitat use, as well as behavioral observations during spring migration in 2016 and 2018. Our overall approach was to compare bird distributions and behavior relative to the availability of macroalgal habitat. All behavior assessments were conducted in 2016. We assessed total abundance relative to macroalgal cover in both 2016 and 2018. All statistical analyses were performed in R version 3.6.0 statistical software [57].

#### Description of spatial scales considered

**Fig 2:**
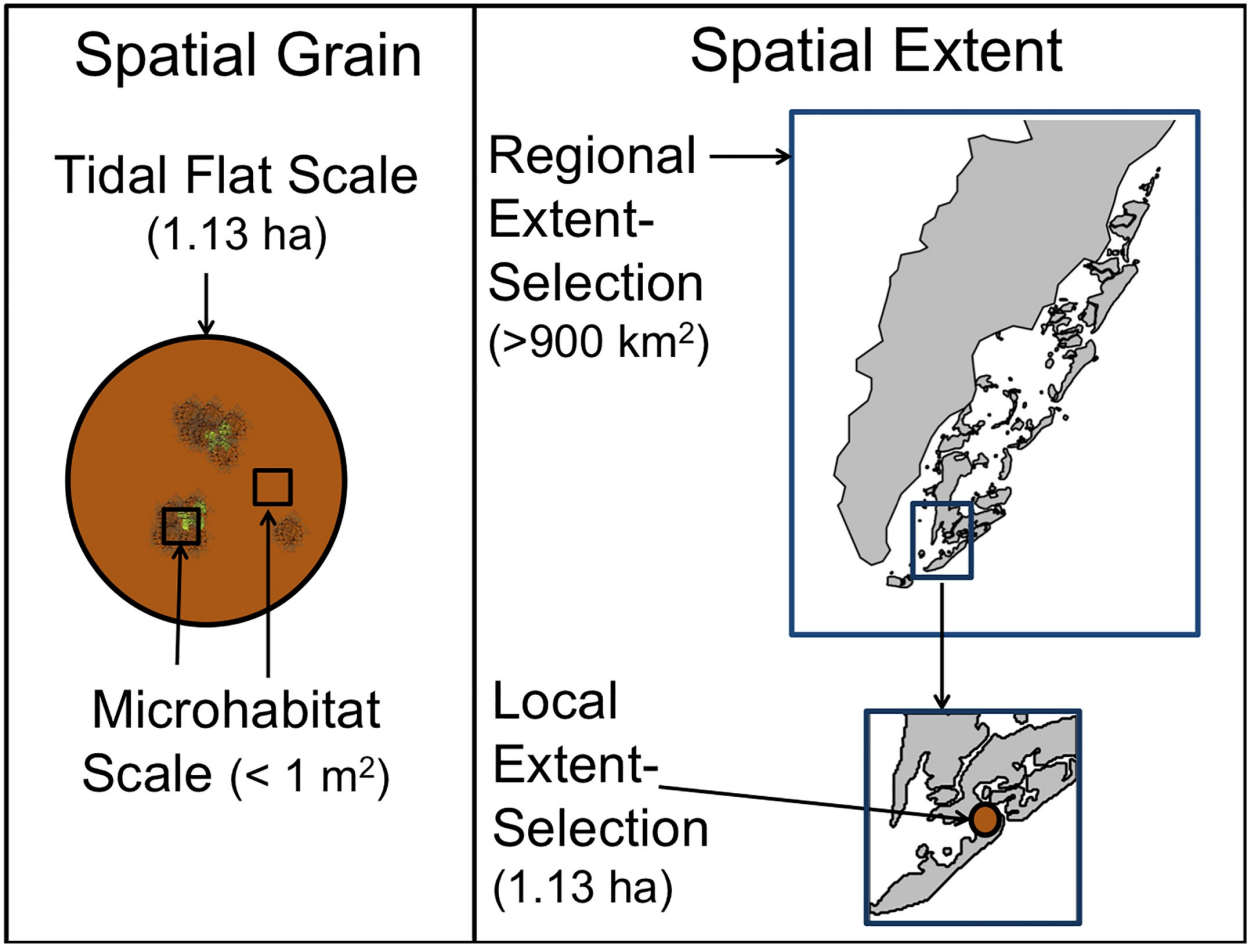
Schematic of spatial scales analyzed. Left side shows spatial grains (tidal flat vs. microhabitat), with the brown circle depicting a circular tidal flat plot. The right side shows spatial extent (regional vs. local) with land in gray and water in white. Relative areas shown in parentheses.

We analyzed selection of macroalgal and bare sediment habitats by shorebirds at multiple spatial scales. To determine how shorebirds respond to macroalgae at different spatial grains, shorebird habitat use was investigated at two scales: “microhabitat” (< one m^2^), and “tidal flat” (~ one ha) (Fig 2). We examined microhabitat scale selection over two spatial extents: “local” and “regional” (Fig 2). Local selection was measured for each tidal flat separately to determine how shorebirds used microhabitat relative to the availability of that microhabitat in their immediate surroundings. Regional-selection compared the use of a microhabitat across the VCR to the availability of that microhabitat across the entire VCR. The basic difference between the local and regional extents was that for local selection we assumed the use of a microhabitat was dependent on tidal-flat-specific characteristics, and the proportional use could differ from one flat to another. Regional selection assumed all tidal flats are equally available and used by shorebirds, i.e. the use of bare substrate on tidal flat A does not differ from the use of bare substrate on tidal flat B. Differences in selection between local and regional selection would suggest the selection of microhabitat is dependent on local factors.

#### Approaches to measure selection

We considered shorebird response to *A*. *vermiculophyllum* by looking at both habitat selection and time budgets. Habitat selection was estimated through surveys of tidal flats that counted and identified shorebirds and their habitat use. We also tested for selection of microhabitats in shorebird foraging behavior by estimating time budgets. Time budgets are a method of quantifying the relative time spent by organisms on different activities [58]. Here, we were interested in comparing the proportion of time spent foraging from bare microhabitat with time spent foraging from macroalgal microhabitat, and comparing those proportions with the availability of microhabitats in the environment. Birds’ time budgets may differ from habitat selection due to interspecific or conspecific interactions, the species foraging specialization mode, or environmental factors. By using these different approaches, we were able to detect the response of shorebirds to *A*. *vermiculophyllum* while accounting for the spatial scale of shorebird habitat selection, spatial heterogeneity among tidal flats, and differences between population spatial distribution and individual behavior.

#### Characterization of macroalgal habitat

To measure macroalgal cover in 2016 we surveyed transects using a 0.25 m^2^ quadrat along three 100 m transects (n = 10 transect^-1^). We estimated macroalgal biomass from three vegetated sites to estimate percent *A*. *vermiculophyllum* by mass using previously described methods [43]. In 2018 we visually estimated macroalgal cover and the percent area submerged for each tidal flat. We collected macroalgal biomass from a single 120 m transect on each plot using a 0.0625 m^2^ quadrat, and identified *A*. *vermiculophyllum* to estimate the percent by mass in the macroalgal community (n = 8). Mean macroalgal cover, biomass, and proportion *A*. *vermiculophyllum* were calculated. We tested the mass of *A*. *vermiculophyllum* for a relationship between macroalgal cover and total macroalgal mass, using simple linear regression. Regressions exhibited heteroscedastic residuals, so a square root transformation was used for total macroalgal cover and mass, and the mass of *A*. *vermiculophyllum*. Models did not violate heteroscedasticity assumptions after transformation. We checked for the effect of outliers by removing potentially influential observations, but did not find that removing these observations changed the results. The distribution of the residuals was moderately not normal, but the non-normality was caused by the presence of zeros in the data set. When these were removed the residuals were normal, and results did not change. Since removing neither outliers nor zeros changed the results, the regression results are presented for the full data set.

#### Invertebrate prey and shorebird predation risk

We collected benthic prey data in 2016 to characterize the prey resources on VCR tidal flats. The prey data were relatively sparse, so are presented as supplemental information (S1 File). To reduce predation risk, which may disproportionately affect the distribution of smaller shorebirds such as peeps and dunlin, we selected tidal flats distant from woody vegetation and forest edges. We still noted the presence of raptors, and ‘vigilant’ behavior in birds that could suggest they were avoiding predators. We only observed raptors or vigilant behavior in two observation in 2016, and never in 2018. Therefore, we assumed predation risk was not an important factor affecting shorebird distributions and behavior in this study.

### Field surveys of shorebirds

#### Field surveys of abundance and habitat use

In 2016 we surveyed nine sites 7–10 times each between April 20-May 31. After excluding surveys conducted while the tidal flat was partially submerged, and observations of birds that were not foraging, our data set included 331 scan surveys. Survey data were averaged by species for each day, at each site, resulting in 986 independent samples of habitat use by focal species. These surveys usually lasted 3 hours (1.5 hours before and after low tide), or for the length of time the tidal flat was exposed (during bad weather and neap tides as short as one hour at some sites, or during spring tides up to 5 hours in a few cases). Surveys were conducted by individual researchers with a spotting scope in the field, and observations were taken on a voice recorder and transcribed to data sheets later. At 15 minute intervals we counted every shorebird on the site from a single scan of the flat, recorded taxa to the lowest level possible, the microhabitat used (tidal channel, bare sediment, macroalgal mat, oyster reef, shallow water, and water-edge) and the bird’s general activity (foraging, searching, roosting/preening, prey handling, aggression) [59]. To ensure we did not double-count birds we began these scans at a designated edge of the flat, and moved our scope as we identified birds across the flat until we had viewed the entire flat. If a flock of birds appeared on the flat before we completed the scan, but landed on a point already counted, we would include those birds. We were careful to notice birds walking in the direction we were scanning so as to not count them twice. Scans usually lasted 5–10 minutes.

From May 11–30, 2018 shorebirds were surveyed using a point count method, and each of the 36 sites was surveyed one time. Point counts involve identifying every bird by either sight or sound in a designated area within a standardized amount of time. We observed 1138 individuals of focal species, and categorized their habitat use. Point counts are a well-established method to capture population dynamics and distributions of mobile species like birds. Surveys were conducted within 3 hours of low tide while flats were exposed. Scans were conducted in the same way they were in 2016, beginning by pointing the spotting scope toward one edge of the flat and slowly moving the scope across the flat until we had identified all of the birds present. In 2018 data were collected by two individuals: one looked through the scope and counted birds and a second researcher recorded the observations on a data sheet. We recorded the microhabitat used by focal species listed in Table 1. Initially, we did not record microhabitat use by whimbrel as we did not expect them to be abundant. However, after identifying them as a species with a high occurrence on mudflats (present on 30% of observed mudflats), we added them as a focal species.

#### Field observations of behavior

We conducted behavioral observations in 2016 to assess how foraging behavior changed relative to tidal flat characteristics. In total, we observed shorebird foraging behavior for 1762 minutes. We conducted focal animal observation in accordance with standard protocols [58,59]. The focal observations were conducted during the same observation periods as the 2016 surveys described above, in between 15-minute scans. Data were again recorded on a voice recorder, and later transcribed to data sheets. Focal foraging observations lasted 3 minutes, or until we lost sight of the bird for more than 30 seconds. After averaging data to species, by day, and by site, we had 376 independent samples to calculate time budgets. We focused on two representative species from each foraging mode: dunlin and willet as the flexible-foragers, and semipalmated plover and black-bellied plovers as the specialized-foragers [59].

During observations we recorded ‘continuous’ behavior, where every action taken by the focal bird is identified and tallied during a designated observation window [59]. Discrete actions were recorded as one of the following: peck, probe, or step. We were not confident in our ability to differentiate between pecks and probes for some birds, especially when the bird was foraging from macroalgae which obscured our view. Therefore, we combined pecks and probes into a ‘foraging-action’ category. We also recorded activity “states” such as preening, roosting, handling, or aggressive interactions.

We also collected ‘instantaneous’ behavior data during these 3-minute focal animal observations. To determine whether shorebirds modified their foraging behavior we constructed time budgets from ‘instantaneous’ behavior data. Every 30 seconds during the observation we recorded the shorebirds behavior and microhabitat use, using the same categories as above [59]. From these instantaneous samples we constructed time-budgets for shorebirds including percent time foraging, searching, handling prey, etc. [59].

### Tidal-flat scale statistical analyses

#### Tidal-flat scale abundance

We statistically compared the percent cover of macroalgae with bird abundance and behavior at the tidal-flat scale. We used generalized linear models (GLM) to test for a relationship between shorebird abundance with macroalgal cover in 2018 [60]. The gradient provided by the nine sites in 2016 was not sufficient to identify a pattern at the tidal-flat scale. We ran tests for each species, for total shorebirds, and for shorebirds grouped as flexible-foragers, and specialized-foragers. Individual species. total shorebird, and flexible-forager abundances all followed a negative-binomial distribution (count data with over-dispersion), while specialized-foragers had lower variance and so a Poisson distribution was appropriate [60]. We checked the model residuals to make sure assumptions were met, and inspected the data for influential outliers. One site appeared it could be overly influential. We re-ran models without it, and found that the model for the abundance of peeps became non-significant after removing it. The results did not change for any other model after removal.

#### Tidal-flat scale behavior

The ‘continuous’ behavior data were used in tidal-flat scale assessments. Rates were calculated as number of discrete actions (steps or foraging-actions) taken per minute. ‘States’ could not be tallied as discrete actions during the continuous observations since these activities (e.g. handling prey) could last for an extended period and include many different types of movements [59]. As a result, we did not calculate rates for preening, roosting, handling, or aggression. Step rate provides a measure of search time at the tidal-flat scale, while foraging-action rate has been shown to correlate closely with prey abundance [22]. We only included observations that lasted at least 30 seconds. Observations were first averaged to species by date, then to foraging mode by date. We then averaged to site to compare rates with macroalgal cover (n = 9). Total observations and independent samples after averaging are reported in the Supporting Information (S1 Table). We compared the step- and foraging-action rates with macroalgal cover using a GLM with a Gamma distribution. Models met all regression assumptions.

### Microhabitat scale analyses

#### Resource selection functions

For the microhabitat scale we considered sub-m^2^ patches as the unit of study and used resource selection functions [36]. Resource selection functions (RSFs) statistically compare the use of a resource (percentage of organisms using a given habitat/resource) with the availability of the resource (percent cover) [36]. We calculated a selection ratio adapted from Manly et al. (2002).

wi=uiutπiπt (1)

The numerator estimates a use ratio where *u*_*i*_ = number of birds using microhabitat *i* for foraging, and *u*_*t*_ = total number of birds seen using any microhabitat for foraging. The denominator estimates habitat availability where *π*_*i*_ = number of units of microhabitat *i*, and *π*_*t*_ = total number of units of microhabitat *i*. We estimated habitat availability as microhabitat percent cover, described above. To investigate how microhabitat selection varied over regional vs. local spatial extents, and between time budgets and spatial habitat selection, this equation was modified and all formulations can be found in the Supporting Information (S2 File). We present results by species and foraging mode. Total comparisons by foraging mode were equal to twelve: 2 measurement types (time budgets and habitat selection) x 2 spatial extents (local and regional) x 2 microhabitats (macroalgal-bare) equal 8 tests in 2016. Added to those were 2 spatial extents (local and regional) x 2 microhabitats (macroalgal and bare) in 2018, equal to 4 tests in 2018. Together these summed to 12 tests in total. For the species level there were 12 tests in total, as described here, for black-bellied plover, semipalmated plover, dunlin, and willet. For dowitchers, peeps, and whimbrel, we did not compute time budgets, so there were 8 tests in total. To determine whether shorebirds used microhabitat differently from availability while accounting for multiple comparisons we used a two-tailed chi-squared goodness-of-fit tests with a Bonferonni correction (α/(2*n) where n = number of tests and α = 0.05) [36]. This approach is valid when there are at least 5 observations for each test [36]. Selection was considered significant if the 95% confidence interval did not overlap one. Confidence intervals were calculated from modified standard error formulas as recommended, and are provided in the Supporting Information (S2 File) [36].

We also tested if selection differed by foraging mode (flexible-foragers differed from specialized-foragers) at each of these levels [36]. This was done with a two-tailed chi-squared goodness-of-fit test as before, by calculating a confidence interval for the difference of the selection ratios [36]. If the interval did not overlap zero, we considered the difference to be significant [36]. We calculated 90% (α = 0.10), and 95% (α = 0.05) confidence intervals, without a Bonferonni correction since comparisons at each scale were considered unique.

### Microhabitat scale time budget RSFs

The ‘instantaneous’ data used to construct time-budgets include the same number and length of independent observations as the ‘continuous’ used in tidal-flat scale behavior analyses (described above). The time-budget selection ratio was calculated as:

$$\frac{f_{i}}{f_{t}}$$ (2)

where *f*_*i*_ is the proportion of time spent foraging on microhabitat *i* and *f*_*t*_ is the total proportion of time spent foraging. Weather conditions, the density of birds, and presence of certain species (gulls can steal food from smaller shorebirds), all may have impacted shorebird behavior during a given day. To control for dependent behavior among individuals, and to avoid more abundant species biasing the statistic, we first calculated a species-specific ratio per site per day, then averaged to foraging mode (S1 Table). Only observations when the tidal flat was completely exposed were included (S1 Table).

### Local time budgets

At the local scale, the proportion of time flexible or specialized-foragers spent foraging from macroalgal or bare microhabitat was compared to the percent cover of microhabitat available on the site where the birds were observed (Eqn. A in S2 File). Since we surveyed 6 sites with > 0% cover in 2016, we calculated a selection index for each observation day, for each foraging mode at each of those 6 sites, and then calculated the overall selection index and confidence interval from those aggregated estimates [36] (Eqns. A and B in S2 File, S1 Table).

### Regional time budgets

At the regional scale, we compared the overall percent time spent foraging from macroalgal or bare microhabitat with the availability of microhabitat available for the entire VCR (Eqns. C and D in S2 File). For the regional case, all 9 sites surveyed in 2016 were considered, average selection was calculated by foraging mode for all sites together, and then compared to average macroalgal or bare cover across those 9 sites to compute the selection ratio and confidence interval (Eqns. C and D in S2 File, S1 Table).

### Microhabitat scale habitat selection RSFs

We used the 36 point-count surveys from 2018, and the surveys from 2016, to assess habitat selection, and independent samples included for each test are detailed in the Supporting Information (S1 Table). We calculated microhabitat use as the proportion of birds observed foraging from microhabitat *i* in the scans. The use ratio took the form:

$$\frac{u_{i}}{u_{t}}$$ (3)

where *u*_*i*_ was the number of individuals observed foraging from microhabitat *i*, and *u*_*t*_ was the total number of individuals observed. The use ratio was calculated separately for specialized and flexible-foragers.

### Local habitat selection

To determine how birds selected for or against microhabitat locally we divided the proportion of birds using microhabitat *i* at each site by the relative availability of microhabitat *i* at each site. This resulted in a selection ratio being computed for each site, so that the sample size was equal to the number of sites sampled (S1 Table). Those ratios were then averaged, and confidence intervals were computed [36] (Eqns. E and F in S2 File).

### Regional habitat selection

For the regional scale we calculated a single selection ratio by foraging mode for the entire VCR. In 2016 we averaged multiple observations collected at a single site to species by site by day, as before (S1 Table). In this case, *u*_*i*_ was the total number of birds observed across all 36 flats in 2018, or 9 flats in 2016, that foraged on microhabitat *i*, *u*_*t*_ was the total number of birds observed on any microhabitat, and the use ratio was divided by the average percent cover of microhabitat *i* across all sites to get the selection ratio and its confidence interval (Eqns. G and H in S2 File).

## Results

### Characterization of macroalgal habitat

Average macroalgal cover at the tidal-flat scale was 12.9% (range: 0–34%; n = 9) and 12.3% (range: 0–75%; n = 36) in 2016 and 2018, respectively. *Agarophyton vermiculophyllum* made up 67.6% of the surveyed macroalgae by biomass in 2016 and 75.4% in 2018. The mass of *A*. *vermiculophyllum* significantly correlated with the percent cover of macroalgae at sites (coefficient = 0.42, R^2^ = 0.72, p-value < .0001), and the total macroalgal mass (coefficient = 1.07, R^2^ = .95, p-value < .0001) (S1 and S2 Figs).

### Species-level analyses

Species abundance, habitat selection, and time budgets within a foraging mode were either statistically significant in the same direction (positive/preference or negative/avoidance), or not significant (Tables 2 and 3). At the tidal flat scale the only species significantly correlated with macroalgal cover was dunlin, which increased in abundance with increasing macroalgal cover (coefficient = 0.07, SE = 0.02, df = 33, z value = 4.61, p < 0.00, Table 2). Each species demonstrated significant selection for microhabitat in habitat selection or time budgets at one or more spatial extents (Table 3). Flexible-forager species (dowitcher, dunlin, peeps, willet) were more likely to prefer macroalgae, and avoid bare substrate (Table 3). Specialized-foraging species (black-bellied plover, semipalmated plover, whimbrel), were more likely to prefer bare substrate, but rarely showed avoidance of macroalgal microhabitat (Table 3). Since species-level analyses were consistent with foraging mode analyses, we present detailed results at the foraging mode level, below.

**Table 2:** Relationships between tidal-flat scale abundance for each species and macroalgal cover.

Foraging Mode	Taxa	df	Estimate	SE	Z	p-value
Specialized						
	Black-bellied Plover	33	0.00	0.02	0.18	0.86
	Semipalmated Plover	34	-0.04	0.02	-1.66	0.10
	Whimbrel	34	-0.05	0.03	-1.58	0.11
Flexible						
	Dowitcher	34	0.05	0.05	1.02	0.31
	**Dunlin**	**33**	**0.07**	**0.02**	**4.61**	**0.00**
	Peeps	32	0.04	0.02	1.54	0.12
	Willet	34	-0.01	0.03	-0.46	0.65

Tests all used generalized linear models with a negative binomial distribution. Model parameters are given for each species, including the model degrees of freedom (df), coefficient estimate for macroalgae as a predictor of abundance, the standard error for the estimate (SE), the Z-statistic, and the p-value. The only species abundance significantly related to macroalgal cover was Dunlin, shown in bold.

**Table 3:** Habitat selection and time budgets of shorebird species.

	Foraging Mode	Taxa	Habitat Selection				Time Budgets	
			Regional		Local		Regional	Local
			2016	2018	2016	2018	2016	2016
Macroalgae								
	Specialized-Foragers	Black-bellied Plover	**—**	1.19± 0.69	**—**	0.46± 0.64	1.17± 2.05	0.49±1.08
		Semipalmated Plover	0.47± 0.62	0.87± 0.74	**—**	0.72± 0.95	0.82± 1.03	1.78**±** 2.02
		Whimbrel	**A0**^N^	**A0**^N^	**A0**^N^	**A0**^N^		
	Flexible-Foragers	Dowitcher	**P 1.89± 0.95**	**P 3.64± 0.73**	0.40± 0.76	2.07± 3.31		
		Dunlin	**P 2.07± 0.42**	**P 3.19± 0.50**	0.94± 0.76	10.3± 15.2	1.89± 1.57	**P 1.62± 0.55**
		Peeps	1.03± 0.52	**P 5.99± 0.86**	2.40± 3.73	0.94± 1.24		
		Willet	**—**	3.26± 3.61	**—**	4.39± 5.24	2.76± 2.95	2.14± 2.55
Bare								
	Specialized-Foragers	Black-bellied Plover	**—**	**P 1.34± 0.13**	**—**	1.42± 0.44	0.87± 0.36	0.75± 0.57
		Semipalmated Plover	1.07± 0.10	**P 1.35± 0.16**	**—**	1.44± 0.46	1.00± 0.17	1.08± 0.16
		Whimbrel	**P 1.14± 0.03**^N^	**P >1**^N^	**P 1.18± 0.05**^N^	**P >1**^N^		
	Flexible-Foragers	Dowitcher	**A0.34± 0.15**	**A 0.34± 0.12**	**A 0.38± 0.33**	**A 0.44± 0.45**		
		Dunlin	**A 0.70± 0.07**	**A 0.73± 0.10**	**A 0.64± 0.29**	**A 0.63± 0.33**	**A 0.68± 0.26**	**A 0.63± 0.17**
		Peeps	0.98± 0.08	**A 0.42± 0.16**	0.79± 0.49	1.46± 0.73		
		Willet	**—**	0.78± 0.71	**—**	**A 0.21± 0.56**	**A 0.36± 0.42**	**A 0.43± 0.46**

Each column shows the selection ratio and 95% CI. Significant selection shown in bold, and letters indicate preference (P) or avoiding (A). Dashes are shown when two few observations were made to calculate a selection ratio. Time budgets were not computed for all species, gray shading indicates species that were excluded.

^**N**^Not statistically significant, but strong evidence of selection. Whimbrel never used macroalgal habitat, RSF statistics could not be formally conducted.

### Tidal-flat scale analyses

#### Tidal-flat scale abundance

**Fig 3:**
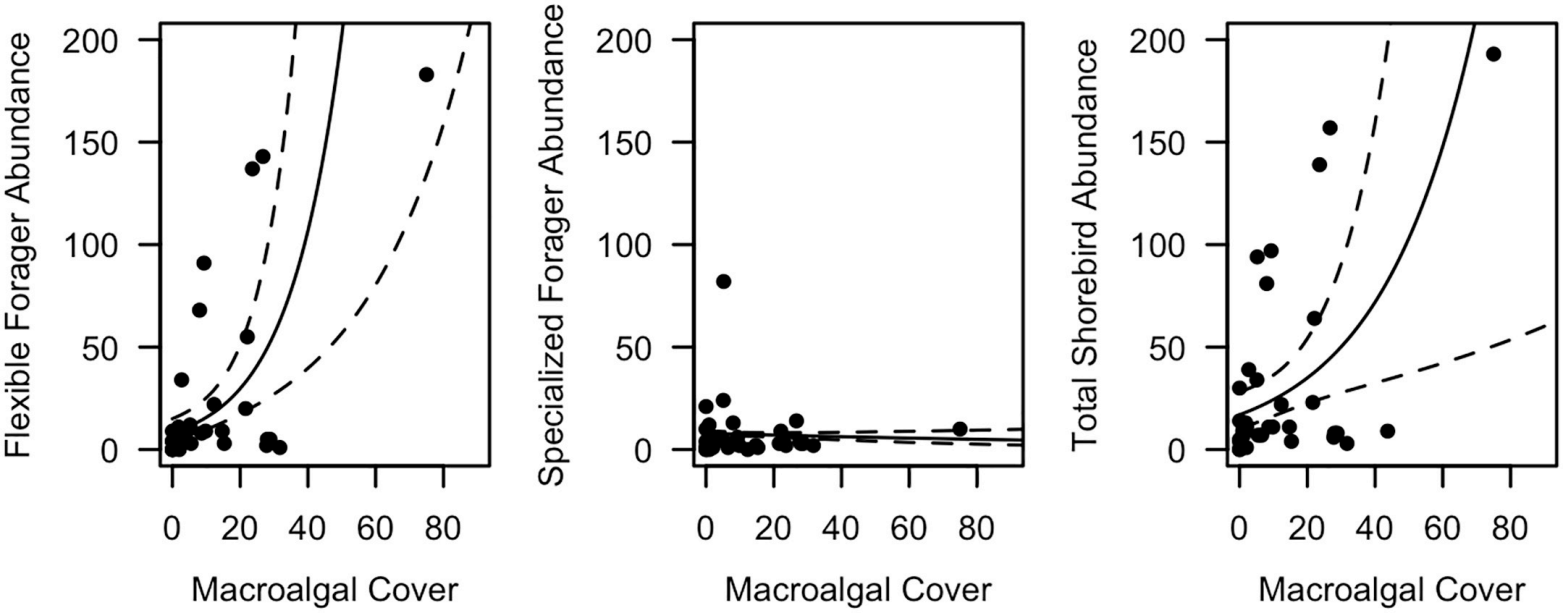
GLM Results for tidal flat abundance in 2018. Plots show regression line and 95% CI between abundance of both foraging modes, and total birds versus macroalgal cover. Flexible-foragers and total birds were positively related to macroalgal cover, while specialized-foragers were not correlated.

Bird abundance for the tidal flat scale is presented for 2018. Average abundance of flexible-foragers was 21 birds ha^-1^ and specialized-forager density was 6 birds ha^-1^ (Table 1). Mean total abundance was 28 birds ha^-1^. Flexible-foraging shorebird abundance was weakly, positively related to macroalgal abundance (coefficient = 0.06, SE = 0.02, df = 32, z value = 4.06, p < 0.001), while specialized-forager abundance was not related to macroalgal cover at the tidal-flat scale (coefficient = -0.01, SE = 0.005, df = 33, z value = -1.25, p = 0.21, Fig 3). Total shorebirds were also weakly positively related to macroalgal abundance (coefficient = 0.04, SE = 0.01, df = 33, z value = 2.82, p = 0.005, Fig 3).

#### Tidal-flat scale behavior

Behavior data are presented for 2016 (only collected that year). Macroalgal cover did not affect foraging action rate or step rate at the tidal-flat scale for either specialized or flexible birds (p > 0.4 for all tests). On tidal flats in the VCR, specialized-foragers took an average of 50 (7 SD) steps min^-1^ and made 8 (2 SD) foraging attempts min^-1^. Flexible-foragers took an average of 55 (11 SD) steps min^-1^ and made 50 (12 SD) foraging attempts min^-1^.

### Microhabitat scale analyses

#### Local time budgets

**Fig 4:**
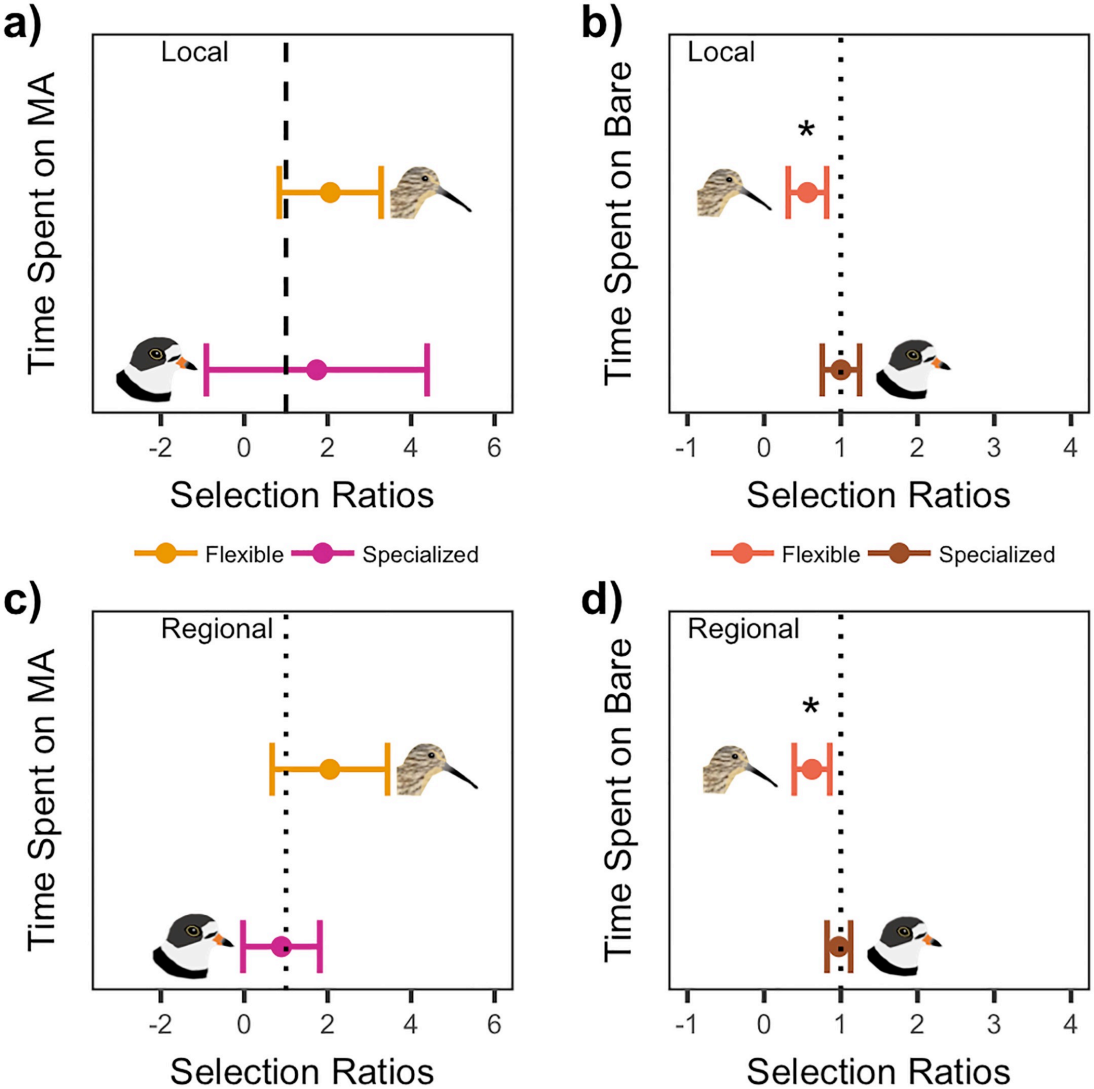
Time budget selection ratios. Selection ratios with 95% CI calculated for time spent on macroalgal habitat (MA), and bare sediment habitat (Bare), by flexible-foraging and specialized-foraging shorebirds. Significant selection (*) when CI does not overlap one (vertical line). Local vs. regional indicates whether selection was computed for the entire study region, or at each individual site. Data for proportion time spent on microhabitats were only collected in 2016, so these plots only show one year of data.

When time budgets were considered locally, neither specialized- nor flexible-foragers appeared to select for or against macroalgae (Fig 4). The time spent foraging from macroalgal substrate at the local scale did not differ by foraging mode (wm,flexible − w_m,specialized_ = 0.32 ± 1.99 CI, Table 4). However, flexible-foragers spent less time on bare microhabitat than would be expected based on local availability, apparently avoiding bare substrate during focal observations (w_b_ = 0.56 ± 0.25 CI, Fig 4). Meanwhile, specialized-foragers spent a proportional amount of time on bare microhabitat relative to its local availability, similar to their use of macroalgal microhabitat (w_b_ = 1.00 ± 0.24 CI). As a result, foraging mode affected the time spent foraging on bare microhabitat, where specialized-foragers used bare substrate more than flexible-foragers (wb,flexible − w_b,specialized_ = -0.44 ± 0.24 CI, Table 4).

**Table 4:** Comparisons between specialized and flexible forager time budgets.

Time Budget Selection				95% Confidence Interval (w_f_-w_s_)	
Microhabitat	Spatial Scale	w_f_-w_s_	SE (w_f_-w_s_)	Lower Bound	Upper Bound
Macroalgal	Regional	**1.11	0.30	0.54	1.69
	Local	0.28	0.93	-1.55	2.11
Bare	Regional	**-0.17	0.04	-0.26	-0.09
	Local	**-0.47	0.13	-0.72	-0.21

Differences in 2016 (only) time budgets between specialized and flexible foragers.

#### Regional time budgets

When considering regional time budget-selection, we found the proportion of time shorebirds used macroalgal substrate for foraging differed by foraging mode, with flexible-foragers using macroalgal substrate more than specialized-foragers (wm,flexible − w_m,specialized_ = 1.16 ± 1.14 CI, Table 4). Although the time spent foraging from macroalgae by flexible and specialized shorebirds differed, we did not detect selection of macroalgae in either foraging mode’s time budget across the VCR (w_m,flexible_ = 2.05 ± 1.38 CI, w_m,specialized_ = 0.89 ± 0.92 CI, Fig 4). Across the VCR flexible-foragers spent less time on bare substrate than expected by its availability (w_b_ = 0.62 ± 0.23 CI, Fig 4). Specialized-foragers did not appear to select for bare substrate in their time budgets at the regional scale (w_b_ = 0.97 ± 0.15). Although, specialized birds still used bare microhabitat for more time than flexible-foragers (wb,flexible − w_b,specialized_ = -0.35 ± 0.19 CI, Table 4).

### Local habitat selection in 2016

**Fig 5:**
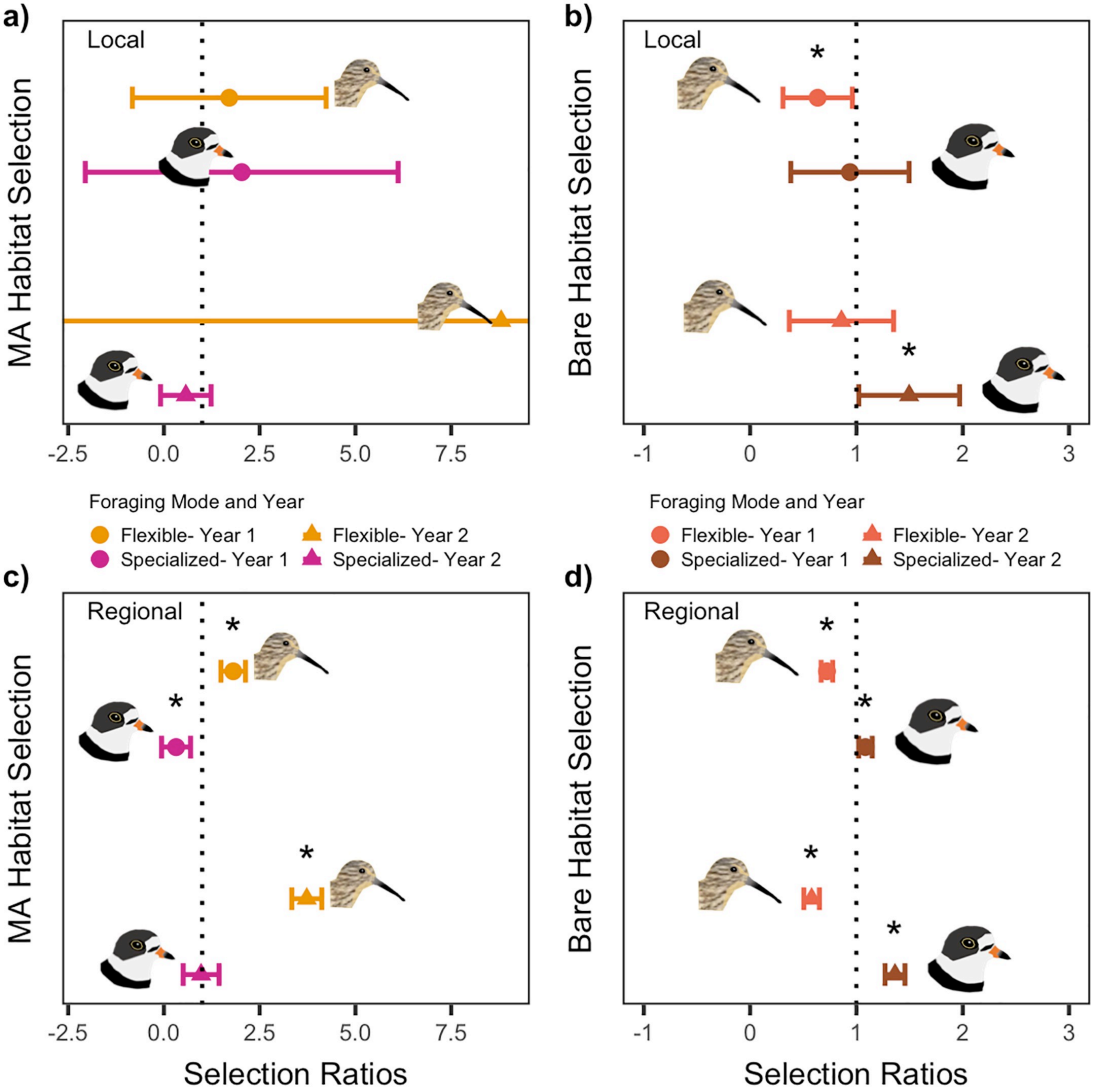
Habitat selection ratios. Selection ratios with 95% CI calculated for selection of macroalgal habitat (MA), and bare sediment habitat (Bare), by flexible-foraging and specialized-foraging shorebirds. Significant selection (*) when CI does not overlap one (vertical line). Local vs. regional indicates whether selection was computed for the entire study region, or at each individual site. Habitat selection data were collected in 2016 (Year 1, circles, n = 9) and 2018 (Year 2, triangles, n = 36). One estimate had a very large CI that extended off the plot (flexible-local-Year 2 = 8.81 ± 13.1).

Locally, flexible and specialized foragers selected macroalgal habitat similarly (95% CI = -3.62 to 2.97, Table 5). Neither group selectively used macroalgae as compared with local availability. Selection of bare microhabitat also did not differ by foraging mode (wb,flexible − w_b,specialized_ = -0.30 ± 0.44, Table 5). But flexible-foragers selectively avoided bare microhabitat at the local scale (w_b_ = 0.64 ± .33 CI, Fig 5). Specialized-foragers used bare habitat in proportion to its local availability on tidal flats (w_b_ = 0.94 ± .56 CI, Fig 5). In 2016, with the exception of flexible-foragers avoiding bare substrate, shorebirds did not show evidence for local selection of either microhabitat.

**Table 5:** Comparisons between specialized and flexible forager habitat selection.

					95% Confidence Interval (w_f_-w_s_)	
Microhabitat	Spatial Scale	Year	w_f_-w_s_	SE (w_f_-w_s_)	Lower Bound	Upper Bound
Macroalgal	Regional	2016	**1.43	0.17	1.10	1.76
		2018	** 2.76	0.21	2.34	3.18
	Local	2016	-0.34	1.68	-3.64	2.95
		2018	*8.23	4.80	-1.17	17.63
Bare	Regional	2016	**-0.36	0.03	-0.42	-0.31
		2018	**-0.78	0.04	-0.87	-0.70
	Local	2016	-0.31	0.23	-0.76	0.13
		2018	**-0.61	0.23	-1.05	-0.16

Difference tests between selection of microhabitat by flexible foragers (w_f_) and specialized foragers (w_s_) at each spatial extent and in both years. SE (w_f_-w_s_) is the standard error of the difference; and 95% CI for the difference is displayed. If the CI did not overlap zero than flexible versus specialized foragers selected habitat differently (**).90% confidence intervals (CIs not shown) indicated by (*).

### Regional habitat selection in 2016

At the regional scale flexible-foragers used macroalgal microhabitat more than specialized-foragers (wm,flexible − w_m,specialized_ = 1.49 ± 0.34 CI, Table 5). Flexible-foragers preferentially used macroalgal microhabitat (w_m_ = 1.81 ± 0.32 CI, Fig 5). Meanwhile, specialized-foragers avoided macroalgal habitat at the scale of VCR (w_m_ = 0.32 ± 0.38 CI, Fig 5). Across the entire VCR flexible-foragers used bare microhabitat less than specialized-foragers (wb,flexible − w_b,specialized_ = -0.36 ± 0.06 CI, Table 5). Bare microhabitat was avoided by flexible-foragers (w_b_ = 0.72 ± 0.05 CI, Fig 5), while specialized-foragers preferred it (w_b_ = 1.09 ± 0.06 CI, Fig 5). Both foraging mode groups selected microhabitat at the regional scale in 2016, and selection differed by foraging mode, with flexible-foragers using macroalgal microhabitat more and bare habitat less than specialized-shorebirds.

### Local habitat selection in 2018

In 2018, local microhabitat selection varied among tidal flats. When we compared selection of macroalgal microhabitat by foraging mode with a 95% CI we did not find a difference (wm,flexible − w_m,specialized_ = 8.23 ± 9.40 CI, Table 5). However, we found that flexible-foragers did use macroalgae locally more than specialized-foragers when compared with 90% confidence intervals (7.89 90% CI). Given the high variability among tidal flats in the proportional use of microhabitats we did not find that either flexible- (w_m_ = 8.81 ± 13.7 CI, Fig 5) or specialized foragers (w_m_ = 0.57 ± 0.66 CI, Fig 5) exhibited significant selection. For bare microhabitat, we found that specialized-foragers used bare microhabitat more than flexible-foragers did at the local-scale (wb,flexible − w_b,specialized_ = -0.64 ± 0.47 CI, Table 5). Specialized-foragers preferentially used bare substrate for foraging locally (w_b_ = 1.50 ± 0.47 CI). But, flexible-foragers again used bare substrate in proportion to its availability locally (w_b_ = 0.86 ± 0.49 CI).

### Regional habitat selection in 2018

Regionally in 2018, populations of shorebirds differed in their selection of macroalgae by foraging mode, with flexible-foragers again preferring to use macroalgal microhabitat more than specialized-foragers (wm,flexible − w_m,specialized_ = 2.76 ± 0.42, Table 5). Across the VCR we found that flexible-foraging shorebirds preferentially used macroalgal microhabitat (w_m_ = 3.73 ± 0.39 CI, Fig 5). While specialized-foragers again used macroalgal microhabitat in proportion to its availability in the VCR (w_m_ = 0.97 ± 0.47 CI, Fig 5). Both foraging modes demonstrated selection with respect to bare microhabitat at the regional scale; and specialized-foragers selected bare substrate significantly more than flexible-foragers (wb,flexible − w_b,specialized_ = -0.78 ± 0.08 CI, Table 5). Specialized-foragers used bare substrate proportionally greater than its availability in the VCR (w_b_ = 1.36 ± 0.09 CI, Fig 5). In contrast, flexible-foragers avoided bare microhabitat at the regional scale (w_b_ = 0.58 ± 0.07 CI, Fig 5).

## Discussion

Specialization was important in mediating the impacts of the exotic *Agarophyton vermiculophyllum* on shorebirds across two years at an important migration stopover. Specialized-foragers, strongly preferred bare, uninvaded microhabitat in their habitat selection, and consistently used bare microhabitat more than flexible-foragers in both time budgets and habitat selection. Flexible-foragers preferred microhabitat with mats of *A*. *vermiculophyllum*, and based on time budgets and habitat selection used macroalgal microhabitat more than specialized-foragers. However, selection was not scale invariant. Shorebirds were the most selective at the microhabitat spatial grain and the regional extent. Local selection of microhabitat, which considered the use of microhabitat on each tidal flat independently, was inconsistent. Considering the tidal-flat spatial grain, shorebirds did not select habitat based on percent cover of macroalgae. Overall, results suggest that an increasingly *A*. *vermiculophyllum* covered coastal zone will favor flexible-foragers, and may negatively affect specialized-foragers, but that the spatial distribution of *A*. *vermiculophyllum* matters.

Most studies examining macroalgal-shorebird interactions have focused on native seaweeds increasing from eutrophication [31,61,62]. A few previous studies have measured the response of shorebirds to *A*. *vermiculophyllum*, but our work represents the first case to our knowledge where specialization has been considered in this system, and only the third case where the response of shorebirds to an invasive macroalga on tidal flats has been studied [4,7]. The consistent response across species within a foraging mode (Table 3) further demonstrates the importance of specialization in foraging mode for determining the response of shorebirds to an invasive macroalga. The most similar study was carried out by Haram et al. 2018, where the response of shorebirds to *A*. *vermiculophyllum* was studied on intertidal flats in a Georgia, USA estuary. Species- and scale- specific responses of shorebirds to *A*. *vermiculophyllum* differed in detail between Haram et al. 2018 and this study. By grouping birds by foraging mode, we revealed that there were greater differences in response to *A*. *vermiculophyllum* than apparent in species-specific analyses, with foraging mode significantly determining if birds preferred or avoided bare and *A*. *vermiculophyllum* microhabitat [7]. Additionally, while Haram et al. found bird abundance increased with *A*. *vermiculophyllum* cover at the tidal flat scale, we observed weak responses by flexible-foragers only when surveying a large number of flats that differed significantly in abiotic characteristics at this scale (Fig 4). Species-specific analyses indicated dunlin likely drove this pattern, suggesting shorebirds do not strongly respond to macroalgae at the tidal-flat scale.

Findings with Haram et al. converge; however, by providing evidence that the response of birds varies with spatial scale, and that certain species prefer *A*. *vermiculophyllum*. Both studies show that flexible-foraging shorebirds (here: dunlin, dowitchers, peeps, willet) prefer *A*. *vermiculophyllum* habitat, while specialized-foragers (here: whimbrel, black-bellied plovers, semipalmated plovers) prefer bare microhabitat, and exhibit mixed responses to *A*. *vermiculophyllum*. Haram et al. found similarly inconsistent results at the local selection scale, with the reason possibly being differences in sediment characteristics and infaunal abundance. Other research has considered the response of shorebirds to native macroalgae, and shorebirds have appeared to change their behavior and abundance inconsistently [31,33,61–63]. Macroalgae that differ in morphology [64,65], and lability [66] will produce different structural changes to bare tidal flats, and host different macroinvertebrate communities. This suggests the response of shorebirds may not only be dependent on foraging mode and spatial scale, but also the macroalgae considered. This variability needs to be resolved in order to predict shorebird responses to invasion and eutrophication driven macroalgal blooms, and focus management and restoration efforts accordingly.

We suggest the strong effect of spatial extent (local vs. regional) and grain (microhabitat vs. tidal flat) on selection by shorebirds can partially explain the variability observed among studies. Specifically, we hypothesize spatial dependency in selection occurs because the net effect of macroalgae on prey availability is context-specific; whereby tidal flat properties such as sediment grain size, and microtopography independently drive macroinvertebrate distributions and accessibility and macroalgae only modify those baseline patterns [67–69]. These tidal flat properties affect sediment penetrability, macrofaunal depth distributions, and densities of different benthic macroinvertebrate guilds [67,70,71]. Thus tidal flat properties affect whether foraging from macroalgal microhabitat is optimal for a shorebird [70–72]. Regional selection in our study was influenced by a subset of flats where large numbers of shorebirds used macroalgae (flexible-foragers on macroalgae per site: 0–183; specialized-foragers: 0–10). Because foraging behavior can be optimized over short timescales shorebirds can respond to the energetic tradeoffs between macroalgal or bare substrate at the microhabitat scale [70–72]. Previous studies have been conducted over smaller regions and more homogeneous environments than the work we have conducted here, suggesting differences among study results is related to the variability in tidal flat properties among these studies [7,31,61,62]. We think it is likely prey density and accessibility was partly controlled by tidal flat properties that varied among published studies. Over entire regions, like the VCR, the selection for or against *A*. *vermiculophyllum* microhabitat should be generalizable for populations of specialized-foragers (prefer bare) and flexible-foragers (prefer *A*. *vermiculophyllum*), but selection at small spatial extents (local) is context-specific.

Neither specialized- nor flexible-foragers strongly selected tidal flats with respect to *A*. *vermiculophyllum* abundance; rather, when preferred tidal flats were vegetated shorebirds partitioned microhabitats according to specialization. Although we did not quantify the prey community in macroalgal microhabitat, previous work indicates lower profit small crustaceans should increase in density, while the accessibility of higher profit surface-deposit feeders should decrease. It appears flexible-foragers target the high density, lower profit resources in macroalgal microhabitat, while specialized-foragers used them occasionally, in keeping with previous observations of foraging shorebirds [7,31,34,72]. Since all of the species studied in this work were dietary generalists, it is unlikely that the difference between specialist- and flexible-forager selection of microhabitat was due to the identity of the prey community present in the substrate. Rather, the distribution of prey sizes, and the accessibility of prey, were probably the most important factors driving the difference.

Generalist species often colonize suboptimal habitat, including that affected by invasive species, and this habitat partitioning can reduce competition [1,8,10]. *A*. *vermiculophyllum* increases habitat complexity on some tidal flats. It also obscures epifaunal movement, covers infaunal burrows, and potentially changes the depth at which preferred prey items are living. As a result, it should alter the accessibility of prey for specialized-foragers that prefer bare microhabitat. However, high variance in food quantity and quality can increase habitat selection in other vertebrates, and has been hypothesized elsewhere to explain shorebird tidal flat-selection [31,73]. High variance in resources can be more important than the overall abundance of food for habitat selection in vertebrates because of neurological perception mechanisms [73]. Since specialized-foragers did not increase or decrease with macroalgal cover at the tidal-flat scale, we hypothesize the heterogeneity in prey accessibility caused by *A*. *vermiculophyllum* compensates for the net decrease in bare microhabitat.

Increasing *A*. *vermiculophyllum* could nonetheless harm specialized-foragers if total cover increases to a point where heterogeneity at the tidal-flat scale is reduced. Macroalgal mats are usually patchily distributed in the VCR, but as *A*. *vermiculophyllum* abundance increases these patches begin to form single homogenous mats covering hundreds of meters of tidal flat. Since obtaining prey directly from *A*. *vermiculophyllum* may not be profitable for visually foraging, specialized-foragers, our results suggest *A*. *vermiculophyllum* increasing above some threshold abundance could result in specialized-foragers not meeting their energetic requirements on vegetated tidal flats. If specialized-foragers are excluded from these habitats as a result, coastal ecosystems could experience functional homogenization which can drive biodiversity loss [10].

We observed eight species of international conservation interest foraging from tidal flats in the VCR during their spring migration, many of which foraged on macroalgal mats at least once (Table 6) [23,25]. Atlantic populations of whimbrel might be declining; consequently, the strong avoidance of macroalgal mats by whimbrel is of conservation concern [74]. All of the species we observed in the VCR were either of moderate or high conservation concern (Table 6) [25]. Given the wide geographic distribution of *A*. *vermiculophyllum* [13], and the significant number of at-risk shorebird species utilizing invaded habitat, our findings should be considered in conservation management (Table 6). If sufficient bare substrate remains available at the regional scale specialized-foragers should be able to find good foraging habitat, and forage on the lower profit prey items in macroalgal mats when it is optimal to do so [34,72]. However, the amount and distribution of bare, high-quality substrate required by specialist shorebirds at Atlantic stopovers is unknown. Further, the potential for spread of *A*. *vermiculophyllum* and increasing total macroalgae is significant [12,13]. Future work needs to focus on the tidal flat properties driving high shorebird use, and how *A*. *vermiculophyllum* modifies those patterns. More detailed ecosystem-scale and individual metabolic studies could clarify the capacity for specialized-foraging species to meet their energetic requirements with remaining bare resources, and/or switching to macroalgal-associated prey. While macroalgae may enhance some tidal flat macroinvertebrates, the long-term conservation of shorebird biodiversity relative to impacts of increasing *A*. *vermiculophyllum* remains uncertain.

**Table 6:** Occurrence table for shorebird species from 2016 and 2018 surveys.

Species	Macroalgal Site	Macroalgal Microhabitat	Conservation Concern Score^1^	Conservation Concern^1^
Spotted Sandpiper	✓		9	Moderate
Semipalmated Plover	✓	✓	10	Moderate
Black-bellied Plover	✓	✓	11	Moderate
Dunlin	✓	✓	11	Moderate
Killdeer	✓		11	Moderate
Ruddy Turnstone	✓	✓	11	Moderate
Sanderling	✓	✓	11	Moderate
Whimbrel	✓		12	Moderate
Red Knot	✓	✓	12	Moderate
Lesser Yellowlegs	✓	✓	13	Moderate; Watch
Golden Plover	✓		13	Moderate
Semipalmated Sandpiper	✓	✓	14	High; Watch
Willet	✓	✓	14	High; Watch
Short-billed Dowitcher	✓	✓	14	High; Watch
American Oystercatcher	✓	✓	15	High, Watch
Marbled Godwit	✓	✓	15	High; Watch
Piping Plover	✓		15	High; Watch
Wilson’s Plover	✓	✓	16	High; Watch

Table includes any shorebird species that was seen on a tidal flat. Check mark indicates that species has been observed at least once on a tidal flat site with macroalgae, or on macroalgal microhabitat. Conservation concern scores calculated from species vulnerability, population trends, distribution, and threats^1^. Scores 1–8 are low concern, 9–13 are moderate concern, 14–20 are high concern. Species are included on the watch list if they have a score of 14 or higher, or 13 with steeply declining populations. None of the species observed in the VCR were low concern [25].

## Supporting information

S1 File Macroinvertebrate prey sampling and data. (DOCX)

S2 File Resource selection function equations. (DOCX)

S1 Table Observation and independent samples (n) for each test. For tidal flat tests only one spatial extent was assessed, so those entries are listed as NA.
(DOCX)

S1 Fig Relationship between invasive *Agarophyton vermiculophyllum*, and native macroalgal biomass. Values are g of dry weight on both axes. Relationship is significant, indicating *A*. *vermiculophyllum* drives macroalgal biomass in the VCR.
(TIFF)

S2 Fig Relationship between invasive *Agarophyton vermiculophyllum*, and native macroalgal cover. Values are g of dry weight on x-axis, and percent cover on y-axis. Relationship is significant, indicating *A*. *vermiculophyllum* drives macroalgal cover in the VCR.
(TIFF)
