# Supplementary material for: Impact of exotic macroalga on shorebirds varies with foraging specialization and spatial scale
Source: PLoS One. 2020 Apr 10;15(4):e0231337. doi: 10.1371/journal.pone.0231337 (PMC7147735; doi:10.1371/journal.pone.0231337)
Supplement: S1 File — (DOCX) [file pone.0231337.s001.docx]

**S1 File. Macroinvertebrate Prey Sampling and Data**

In 2016 we collected benthic cores (15.24 cm i.d., 10 cm depth) from three bare sites (n = 3 cores) and three macroalgal sites (n = 6; 3 macroalgal, 3 bare cores). Macrofauna larger than 500-μm were collected, fixed and stored in 90% ethanol until identification. Benthic fauna were identified to coarse taxonomic levels including: small crustaceans (mostly amphipods with few isopods), gastropods (Tritia obsoleta dominant), polychaetes, bivalves, and crabs (fiddler and mud crabs). Biomass was estimated using allometric relationships [52,53]. Individuals from the cores were combined by microhabitat at each site, so that we had 1 independent sample per microhabitat per site. We calculated means and standard deviations for density and biomass of prey by taxonomic group.

Table of macroinvertebrate prey data collected in 2016. At the tidal flat scale three sites with macroalgae, and three sites with no macroalgae were compared. At the microhabitat scale cores collected from macroalgal vs bare substrate across all six sites samples were compared. Ash-free dry weight (AFDW) was lower than we could measure (only a few individuals were collected, then normalized to m^2^ area). Gray shading is displayed for those table entries.

|  |  |  | Density (no. m^-2^) |  | Biomass (g AFDW m^-2^) |  |
| --- | --- | --- | --- | --- | --- | --- |
|  |  |  | Mean | SD | Mean | SD |
| Tidal Flat |  |  |  |  |  |  |
|  | Macroalgae |  |  |  |  |  |
|  |  | Total | 1401.67 | 306.65 | 8710.60 | 1733.01 |
|  |  | Bivalves | 63.99 | 50.90 |  |  |
|  |  | Crabs | 15.24 | 13.96 |  |  |
|  |  | Gastropods | 566.76 | 264.47 | 6203.26 | 2493.84 |
|  |  | Polychaetes | 353.46 | 101.11 | 2322.24 | 685.68 |
|  |  | Small crustaceans | 402.22 | 233.42 | 185.10 | 131.02 |
|  | Bare |  |  |  |  |  |
|  |  | Total | 1614.97 | 1876.30 | 8074.26 | 6432.81 |
|  |  | Bivalves | 0 | 0 |  |  |
|  |  | Crabs | 30.47 | 21.11 |  |  |
|  |  | Gastropods | 274.24 | 247.32 | 4060.56 | 3565.32 |
|  |  | Polychaetes | 1279.79 | 1673.54 | 4002.51 | 3495.72 |
|  |  | Small crustaceans | 30.47 | 21.11 | 11.20 | 12.83 |
| Microhabitat |  |  |  |  |  |  |
|  | Macroalgae |  |  |  |  |  |
|  |  | Total | 1572.31 | 454.87 | 6608.87 | 2310.67 |
|  |  | Bivalves | 109.70 | 101.79 |  |  |
|  |  | Crabs | 24.38 | 27.93 |  |  |
|  |  | Gastropods | 365.65 | 48.37 | 4075.31 | 1802.88 |
|  |  | Polychaetes | 298.62 | 110.20 | 2181.81 | 1182.22 |
|  |  | Small crustaceans | 773.97 | 459.01 | 351.76 | 237.57 |
|  | Bare |  |  |  |  |  |
|  |  | Total | 1423.00 | 1234.34 | 9443.297 | 5220.46 |
|  |  | Bivalves | 9.14 | 10.01 |  |  |
|  |  | Crabs | 18.28 | 20.03 |  |  |
|  |  | Gastropods | 521.06 | 475.45 | 6195.89 | 4454.75 |
|  |  | Polychaetes | 844.05 | 1162.55 | 3232.58 | 2370.46 |
|  |  | Small crustaceans | 30.47 | 35.95 | 14.83 | 22.14 |
