# Supplementary material for: Impact of exotic macroalga on shorebirds varies with foraging specialization and spatial scale
Source: PLoS One. 2020 Apr 10;15(4):e0231337. doi: 10.1371/journal.pone.0231337 (PMC7147735; doi:10.1371/journal.pone.0231337)
Supplement: S2 File — (DOCX) [file pone.0231337.s002.docx]

**S2 File:** Resource selection function equations and sample sizes

N = # tidal flats sampled

B= # independent focal bird observations (1 per species, per site, per day)

π_i_ = sampled units of microhabitat i at a tidal flat

π_t_ = total sampled microhabitat units at a tidal flat

p_i_= (π_i_ / π_t_); proportion of available microhabitat i at a sampled tidal flat

P_i_= (Σ (π_i_ / π_t_)/N); proportion of available microhabitat i in study region

u_i_= number of birds using microhabitat i on a sampled tidal flat

u_t_ = number of birds on a sampled tidal flat

U_i_ = Σ u_i_; total number of birds counted in region using microhabitat i

U_t_ = Σ u_t_; total number of birds counted in region

$\lambda_{i}$ = (u_i_/u_t_) ; proportion of birds using microhabitat i on a sampled tidal flat

$\Lambda$_i_ = (U_i_/U_t_); proportion of birds using microhabitat i in study region

f_i_ = number of times individual bird observed foraging on microhabitat i on sampled tidal flat; averaged so 1 value per species, per day, per site

f_t_ = number of times individual bird observed foraging on sampled tidal flat; averaged so 1 value per species, per day, per site

F_i_ = Σ f_i_; total observations of birds foraging while on microhabitat i across study region

F_t_ = Σ f_t_; total observations of birds foraging across study region

t_i_= (f_i_/f_t_) ; proportion of time spent on microhabitat i at a sampled site

T_i_ = (F_i_/F_t_); proportion of time spent on microhabitat i in study region

Equation A. Local-scale, behavioral selection ratio for time spent on microhabitat.

$$w_{i}= \sum\frac{\left( \frac{t_{i}}{p_{i}} \right)}{B}$$

Equation B. Local-scale, behavioral selection standard error of the ratio.

$$SE\left( w_{i} \right)=\frac{sd(w_{i})}{\sqrt{B}}$$

Equation C. Local-scale, habitat selection ratio.

$$w_{i}= \sum\frac{\left( \frac{\lambda_{i}}{p_{i}} \right)}{N}$$

Equation D. Local-scale, habitat selection standard error of the ratio.

$$SE\left( w_{i} \right)=\frac{sd(w_{i})}{\sqrt{N}}$$

Equation E. Regional-scale, behavioral selection ratio for time spent on microhabitat.

$$w_{i}= \frac{T_{i}}{P_{i}}$$

Equation F. Regional-scale, behavioral selection standard error of the ratio.

$$SE\left( w_{i} \right)= \sqrt{T_{i} \times\left( \frac{1-T_{i}}{F_{t}{P_{i}}^{2}} \right)}$$

Equation G. Regional-scale, habitat selection ratio.

$$w_{i}= \frac{\Lambda_{i}}{P_{i}}$$

Equation H. Regional-scale, habitat selection standard error of the ratio.

$$SE\left( w_{i} \right)= \sqrt{\Lambda_{i} \times\left( \frac{1-\Lambda_{i}}{U_{t} {P_{i}}^{2}} \right)}$$
