# Supplementary material for: Impact of exotic macroalga on shorebirds varies with foraging specialization and spatial scale
Source: PLoS One. 2020 Apr 10;15(4):e0231337. doi: 10.1371/journal.pone.0231337 (PMC7147735; doi:10.1371/journal.pone.0231337)
Supplement: S1 Table — For tidal flat tests only one spatial extent was assessed, so those entries are listed as NA. (DOCX) [file pone.0231337.s003.docx]

Table S1. Observation and independent samples (N) for each test. For tidal flat tests only one spatial extent was assessed, so those entries are listed as NA.

| Spatial Grain | Variable | Year | Spatial Extent | Foraging Mode | Observations | | N |
| --- | --- | --- | --- | --- | --- | --- | --- |
| Tidal Flat | Abundance | 2018 | NA | Flexible | NA | | 34 |
|  |  |  |  | Specialized | NA | | 35 |
|  | Behavior | 2016 | NA | Flexible | | 371 | 9 |
|  |  |  |  | Specialized | 334 | | 9 |
| Microhabitat | Habitat Selection | 2016 | Local | Flexible | 3566 | | 6 |
|  |  |  |  | Specialized | 347 | | 6 |
|  |  |  | Regional | Flexible | 4572 | | 851.2 |
|  |  |  |  | Specialized | 1171 | | 135.3 |
|  |  | 2018 | Local | Flexible | NA | | 28 |
|  |  |  |  | Specialized | NA | | 27 |
|  |  |  | Regional | Flexible | NA | | 878 |
|  |  |  |  | Specialized | NA | | 260 |
|  | Time Budgets | 2016 | Local | Flexible | 87 | | 35 |
|  |  |  |  | Specialized | 60 | | 28 |
|  |  |  | Regional | Flexible | 134 | | 50 |
|  |  |  |  | Specialized | 133 | | 59 |
|  |  |  |  |  |  | |  |
