# Supplementary figures and images for: Impact of exotic macroalga on shorebirds varies with foraging specialization and spatial scale
Source: PLoS One. 2020 Apr 10;15(4):e0231337. doi: 10.1371/journal.pone.0231337 (PMC7147735; doi:10.1371/journal.pone.0231337)

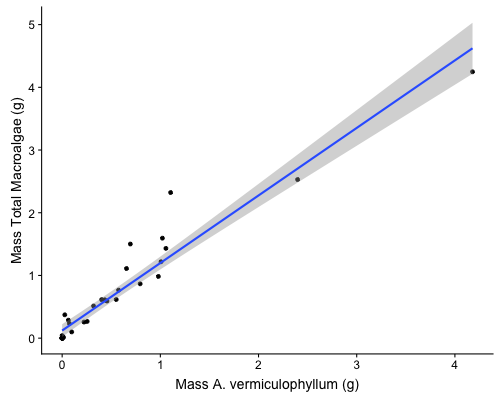

Supplement: S1 Fig — Values are g of dry weight on both axes. Relationship is significant, indicating A. vermiculophyllum drives macroalgal biomass in the VCR. (TIFF) [file pone.0231337.s004.tiff]

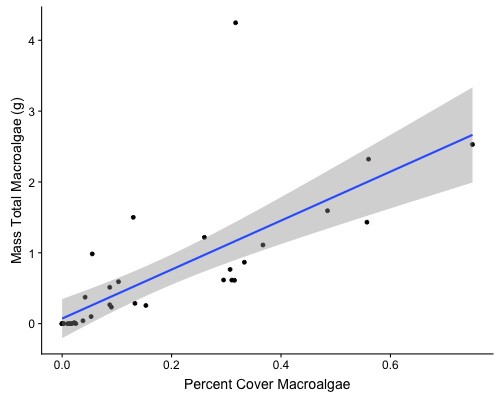

Supplement: S2 Fig — Values are g of dry weight on x-axis, and percent cover on y-axis. Relationship is significant, indicating A. vermiculophyllum drives macroalgal cover in the VCR. (TIFF) [file pone.0231337.s005.tiff]
